# Proof of lung muscarinic receptor occupancy by tiotropium: Translational Positron Emission Tomography studies in non-human primates and humans

**DOI:** 10.3389/fnume.2022.1080005

**Published:** 2023-01-18

**Authors:** Zsolt Cselényi, Aurelija Jucaite, Pär Ewing, Per Stenkrona, Cecilia Kristensson, Peter Johnström, Magnus Schou, Martin Bolin, Christer Halldin, Bengt Larsson, Ken Grime, Ulf G Eriksson, Lars Farde

**Affiliations:** ^1^ PET Science Centre, Precision Medicine and Biosamples, R&D, AstraZeneca AB, Stockholm, Sweden; ^2^ Department of Clinical Neuroscience, Centre for Psychiatry Research, Karolinska Institutet and Stockholm County Council, Stockholm, Sweden; ^3^ DMPK, Research & Early Development, Respiratory & Immunology, BioPharmaceuticals R&D, AstraZeneca AB, Gothenburg, Sweden; ^4^ Clinical Development, Research & Early Development, Respiratory & Immunology, BioPharmaceuticals R&D, AstraZeneca AB, Gothenburg, Sweden

**Keywords:** positron emission tomography, [^11^C]VC-002, lung, muscarinic receptors, receptor occupancy, tiotropium

## Abstract

**Introduction:**

Molecular imaging has not been used to support the development of drugs for the treatment of pulmonary disorders. The aim of the present translational study was to advance quantitative pulmonary PET imaging by demonstrating occupancy of the reference asthma drug tiotropium at muscarinic acetylcholine receptors (mAChR).

**Methods:**

PET imaging was performed using the muscarinic radioligand [^11^C]VC-002. The key methodological step involved estimating muscarinic receptor binding while disentangling it from the background of non-specific binding. The relationship between tiotropium exposure and receptor occupancy (RO) was assessed in non-human primates (NHPs) after intravenous injection of tiotropium doses at a broad dose interval (0.03–1 *µ*g/kg). The feasibility of measuring RO in the human lung was then confirmed in seven healthy human subjects after inhalation of a single therapeutic dose of tiotropium (18 *µ*g).

**Results:**

There was an evident effect of tiotropium on [^11^C]VC-002 binding to mAChRs in lungs in both NHPs and humans. In NHPs, RO was 11 to 78% and increased in a dose dependent manner. Non-displaceable binding in NHPs was about 10% of total binding. In humans, RO was 6%–65%, and non-displaceable binding was about 20% of total binding at baseline.

**Discussion:**

The results demonstrate that [^11^C]VC-002 binds specifically to mAChRs in the lungs enabling the assessment of RO following administration of muscarinic antagonist drugs. Furthermore, the methodology has potential not only for dose finding and comparison of drug formulations in future applied studies, but also for evaluating changes in lung receptor distribution during disease or in response to therapy.

**Clinical Trial Registration:**

ClinicalTrials.gov, identifier: NCT03097380.

## 1. Introduction

Muscarinic receptor (mAChR) antagonists together with other bronchodilators are the current mainstay for relieving dyspnoea in patients with chronic lung disorders (1–3). The clinical efficacy of the first long-acting mAChR antagonist (LAMA) tiotropium has over the years spurred development of new LAMAs (4). However, despite their widespread use, the extent and duration of LAMA binding to receptors in human lung tissue *in vivo* is still poorly understood (5).

Receptor occupancy (RO) refers to the percentage of a receptor population that is occupied by a drug targeting the receptors at a specified dose or concentration in plasma. The RO concept was early introduced in positron emission tomography (PET) studies of drugs used to treat neuropsychiatric disorders (6, 7). It serves as a correlate of pharmacodynamic and safety parameters, thereby supporting selection of optimal dose strength and dosing interval to be examined in clinical trials (8–12).

Molecular imaging of mAChRs in the lungs was enabled by early development of radioligand [^11^C]VC-002, a non-selective mAChR antagonist that has been shown to have specific binding to the lung muscarinic receptors(13, 14). Using the radioligand [^11^C]VC-002 we recently performed a PET study in nonhuman primates (NHP) and compared lung mAChR occupancy after inhalation and iv infusion of the LAMA ipratropium (15). The study showed that it is possible to demonstrate a dose-occupancy relationship in lung and demonstrated the therapeutic advantage of the inhaled route of drug delivery. [^11^C]VC-002 binding to muscarinic receptors has recently been further examined in a test-retest study in humans (16). It was concluded that the kinetic behaviour and the binding characteristics of the radioligand appears suitable for applied studies on muscarinic receptor occupancy in the human lungs.

The aim of the present translational PET study was to estimate occupancy of tiotropium at mAChR in the lungs *in vivo*. The relationship between the tiotropium exposure and receptor occupancy was assessed in NHPs after intravenous injection of doses covering a broad range. The feasibility of measuring mAChR occupancy was then confirmed in a small sample of healthy human subjects after inhalation of a single therapeutic dose of tiotropium.

## 2. Materials and methods

### 2.1. Study in non-human primates

The study was approved by the Animal Research Ethical Committee of the Northern Stockholm Region. Three adult female cynomolgus monkeys (mean weight 6.1 kg) were included. The non-human primates (NHPs) were owned by the Centre for Psychiatry Research, Department of Clinical Neuroscience, Karolinska Institutet, and housed in the Astrid Fagraeus Laboratory, Karolinska Institutet, Solna, Sweden.

#### 2.1.1. Study design

The study in NHP comprised of 8 experimental days. Two PET-measurements with [^11^C]VC-002 were performed on each day. A baseline PET-measurement was followed by a second measurement 2.5 h later either at baseline conditions (test-retest) or after i.v. administration of tiotropium (pretreatment). Two of the monkeys (NHP1 and NHP2) participated in the test-retest measurements. All three monkeys (NHP1-3) participated in two pretreatment sessions each. In the pretreatment measurements (*n* = 6), tiotropium was infused over 15 min starting 20 min before the [^11^C]VC-002 injection (for details see Supplementary Figure S1). The six doses of tiotropium varied from 0.03 to 1.0 *μ*g/kg. The highest doses were expected to induce saturating levels of tiotropium binding in lungs. Such conditions were feasible as higher doses were well tolerated in NHP.

#### 2.1.2. Measurement of tiotropium plasma concentration

At each PET measurement with active drug, venous blood samples were collected for determination of tiotropium plasma concentration. The samples were drawn before tiotropium administration at approximately 35 min before [^11^C]VC-002 injection, and after tiotropium administration at 0.5, 1, 5, 15, 30 and 60 min after [^11^C]VC-002 injection. The plasma concentration of tiotropium was measured by solid phase extraction, followed by high-performance liquid chromatography and atmospheric pressure chemical ionization tandem mass spectrometry (LC-MS/MS) (17). The lower limit of quantification (LLOQ) was 5 pM. The area under the plasma concentration curve for the time of the PET measurement (90 min) was calculated and divided by the duration to obtain the average plasma concentration during PET.

#### 2.1.3. PET measurements

General anesthesia was induced by intramuscular injection of ketamine hydrochloride (approximately 10 mg/kg) and after endotracheal intubation maintained by administration of a mixture of sevoflurane (2%–8%), oxygen, and medical air. Head immobilization and safety monitoring procedures (body temperature, heart rate, blood pressure, fluid balance) were performed as described previously (15).

The radioligand [^11^C]VC-002 was prepared at the PET center at Karolinska University Hospital as previously described (13, 15). In each PET measurement, a sterile physiological, phosphate-buffered (pH 7.4) saline (PBS) solution containing [^11^C]VC-002, in a volume not exceeding 5 ml, was injected as a bolus into a sural vein during 5 s. PET data acquisition started at time of the bolus injection. In all measurements the radiochemical purity of [^11^C]VC-002 exceeded 99% at time of injection. The mean radioactivity injected was 154 MBq (SD ± 9 MBq, range 139–172 MBq, *N* = 16 measurements). The molar activity at time of injection was 689 GBq/*µ*mol (SD ± 814 GBq/*µ*mol, range 129–3,195 GBq/*µ*mol) corresponding to an injected mass of 0.25 *μ*g (SD ±18 *μ*g, range 0.025–0.57 *μ*g).

PET measurements were conducted using the high-resolution research tomograph (HRRT) (Siemens Molecular Imaging). Radioactivity in lungs was measured continuously over 63 min using an imaging protocol described in detail previously (15). In short, a transmission scan of 6 min using a single ^137^Cs source was performed immediately before [^11^C]VC-002 injection. Data were acquired continuously in list mode for 63 min after i.v. injection of [^11^C]VC-002. Images were reconstructed for a series of time frames (9 × 10 s, 2 × 15 s, 3 × 20 s, 4 × 30 s, 4 × 1 min, 4 × 3 min, and 7 × 6 min).

#### 2.1.4. Blood sampling for the measurement of radioactivity

Venous blood samples (1–3 ml) were obtained manually at 1, 2, 3, 5, 15, 30, 45 and 60 min for the measurement of radioactivity in whole blood and plasma using a well-counter. The procedure has previously been described in detail (15).

### 2.2. Study in humans

#### 2.2.1. Subjects and study design

The study was approved by the Regional Ethics Committee in Stockholm and the Radiation Protection Committee at the Karolinska University Hospital, Stockholm. Written informed consent was obtained from each subject.

Seven male subjects, 20–50 years of age, were recruited and studied at the PET Centre, Department of Clinical Neuroscience, Karolinska Institutet, Stockholm, Sweden. Subjects were healthy according to medical history, clinical examination, and routine laboratory blood and urine tests. No medications were allowed at time of the study.

The PET-measurements were carried out on a whole-body GE Discovery 710 PET/CT-system at the Department of Nuclear Medicine, Karolinska University Hospital, Solna. Each subject participated in two or three PET examinations that were performed on separate days.

In part 1, subjects H1–H3 first participated in a baseline (BL) measurement. A second PET measurement was performed 7–12 days later and 2 h after inhalation of a therapeutic dose of tiotropium (18 *µ*g).

In part 2, each of four subjects H4–H7 participated in three PET measurements. The first PET (BL) was followed by two PET measurements, each one after pretreatment with tiotropium (18 *µ*g). The pretreatment PET measurements started 30 min after inhalation of tiotropium in subjects H4–H5 and 2 h after inhalation in subjects H6–H7 (for details see Supplementary Figure S1). In both study parts, adverse events were monitored on experimental days and up to one week after the last measurement *via* a follow-up telephone call.

#### 2.2.2. Measurement of tiotropium plasma concentration

In part 1 (subjects H1–H3) the concentration of tiotropium in plasma was not measured. In part 2 (subjects H4–H7), 3 venous blood samples were obtained at start, middle time and end of the PET measurement, i.e., 30–90 or 120–180 min post inhalation of tiotropium. A standard LC-MS/MS analysis of tiotropium concentration in venous plasma was performed, i.e., the same as in NHP (17). The lower limit of quantification (LLOQ) was 1.3 pM (note: lab equipment was different than the one used for NHP samples). The average concentration during PET acquisition was obtained by calculating the area under the plasma concentration curve and dividing it by the duration of PET.

#### 2.2.3. PET/CT measurements

PET examinations, radiochemistry, blood sampling, image processing, and quantification were performed essentially as described in detail in our previous publication on the test-retest reliability of [^11^C]VC-002 binding in the human lung (16). The radiochemical purity of [^11^C]VC-002 exceeded 99% at time of injection in all measurements. The mean injected radioactivity was 220 MBq (SD ± 37 MBq, range 165–296 MBq, *N* = 18) and the molar activity was 398 GBq/*µ*mol (SD ± 250 GBq/*µ*mol, range 51–955 GBq/*µ*mol). The corresponding mass of the radioligand injected was 0.34 *μ*g (SD ± 0.34, range 0.08–1.24 *μ*g).

At time of imaging examination each subject was positioned in the PET/CT system head-first, supine with the chest located within the 16 cm field of view. Initially, a low-dose CT was obtained for the chest. After i.v. injection of [^11^C]VC-002, radioactivity was acquired in list mode for 63 min providing a reconstructed 4D PET image with 33 timeframes with equivalent frame timings as for the NHP study.

The PET image was evaluated and, if possible, corrected for inter-frame subject movements as described previously (16). In short, the reconstructed 4D PET image was frame-by-frame realigned to have a consistent subject position throughout. In case of mismatch of the PET frame and the CT scan used for attenuation correction, the PET image was re-reconstructed to minimize this effect of a movement artifact, by ensuring the correct alignment of the attenuation map for each time frame. The final corrected image was quality checked for the presence of potential residual artifacts related to severe intraframe subject movement.

#### 2.2.4. Blood sampling for measuring [^11^C]VC-002 radioactivity concentration

Before PET an arterial cannula was inserted into one of the radial arteries. After injection of [^11^C]VC-002, 3 arterial blood samples (2 ml each) were drawn manually at 10, 25, and 45 min for the purpose of determining the average plasma-to-whole-blood ratio.

#### 2.2.5. Regions of interest delineation

ROIs for the lungs and the arch of the aorta were delineated automatically as described previously (16). In short, the method relied on the co-registered CT images to identify voxels in the body with a density below water based on the CT Hounsfield unit value. The binary image of such voxels was then refined using the summation PET image and image processing morphological operations to arrive at identifying a ROI covering both lungs. Voxels falling within the volume of the arch of the aorta were identified based on the voxels' early time course of high radioactivity after iv. injection. In addition, the search was restricted to the mediastinal space, and distinguished between venous (e.g., vena cava) and arterial voxels based on the relative timing of peak radioactivity. Finally, the ROIs were applied to the series of PET images to obtain TACs for the whole lung (bilateral) and aorta, respectively.

### 2.3. Quantification of radioligand binding and receptor occupancy

#### 2.3.1. Quantification of [^11^C]VC-002 binding in lungs

The total (i.e., parent plus radioactive metabolites) plasma radioactivity concentration was used as the input function for quantification of binding as previously established and described (15, 16). To derive this curve, the TAC for the aorta was multiplied with the average plasma-to-whole-blood radioactivity ratio calculated from the drawn blood samples as described above.

The parameter used to express [^11^C]VC-002 binding was the total volume of distribution (V_T_), which is an index of total binding in tissue, i.e., the sum of non-specific and receptor-specific binding. V_T_ is numerically equivalent to the tissue partition coefficient, i.e., the ratio of tissue to plasma concentration in case of proper steady state conditions.

The quantification was carried out for each volume element (voxel) using data-driven estimation of parametric images based on compartmental theory (DEPICT), as described previously (16, 18). The range of exponents, employed in the calculation of the table of kinetic basis functions used by DEPICT, was between 0.0136 1/min and 0.6 1/min [as per (18)]. The main output of DEPICT was voxel-wise parametric images of V_T_ in the lungs.

#### 2.3.2. Quantification of receptor occupancy

Receptor occupancy calculation is straightforward when the availability of a reference region with negligible binding provides an estimate of non-specific binding and thus allows for differentiation between specific and non-specific binding in the target region. However, in pulmonary imaging there is no reference region and the specific binding component of a radioligand such as [^11^C]VC-002 cannot be directly quantified (16). The estimation of receptor occupancy following drug administration was instead estimated using the Lassen plot, an established indirect approach (19, 20).

The indirect Lassen approach is based on the total binding (V_T_) values and relies on the assumptions that various parts of the lungs have different levels of mAChR expression, and thus specific [^11^C]VC-002 binding; and that the background of non-specific binding as well as drug exposure at the target is similar across the organ. Based on these assumptions, lung V_T_ data from baseline and pretreatment conditions were entered into a graphical evaluation (Lassen plot), which provided estimates of receptor occupancy as well as the level of non-specific (non-displaceable) binding, V_ND_ (19–21).

The scatter points for the Lassen plot analysis were obtained from the parametric images of V_T_ in lung tissue, following a downsampling procedure to reduce voxel-wise variation of V_T_ estimates, and, consequently, the spread (variance) of the scatter points in the plot. In detail, the voxel count in the parametric images was reduced by a factor of 4 along each dimension, i.e., a 64-fold reduction in the total number of voxels, by calculating the mean V_T_ estimate for each adjacent 4 × 4 × 4 cube of voxels. Then the voxel-wise V_T_ estimates within the lungs at baseline and at pretreatment were extracted and projected into the Lassen plot. For this purpose, the lung ROI mask, obtained at the original PET image resolution, was first eroded to discard voxels at the edge of the lungs (within a 2-voxel margin from the surface), and then downsampled in two steps. Each downsampling step reduced the volume size by a factor of 2 along each dimension, i.e., a 8-fold reduction in the total count of voxels, and then retained voxels as “within-lung” which had a downsampled value >0.5 (i.e., > 50% probability for lung). The final downsampled lung ROI mask had the same resolution as the downsampled V_T_ parametric image and could thus be applied for the extraction of voxel-wise lung V_T_ values.

Lassen's original graphical approach entailed plotting the difference in V_T_ between baseline and pretreatment conditions vs. the baseline V_T_ values across lungs in a scatter plot. Receptor occupancy was estimated by performing a constrained, weighted linear fit that gave more weight to scatter points in the Lassen plot with a higher local kernel density. A modified variant of the Lassen approach was used in one subject (H6), which, in lack of usable baseline data, relied on V_T_ data from only one pair of experiments following drug inhalation as well as the measured average drug concentrations in plasma during PET according to a previously described methodology using linear regression analysis (21).

The presently obtained receptor occupancy values were compared to the previously reported test-retest study in humans (16). For that purpose, the test-retest dataset was re-analyzed using the same settings as in the present study, i.e., parametric images were obtained using only the first 63 min of the acquired 90-min data and with the same DEPICT settings as specified above in section 2.3.1. To obtain an unbiased apparent occupancy estimate in the test-retest dataset, the Lassen plot analysis was performed twice, switching the order of the test and retest datasets, and the mean slope was then calculated.

#### 2.3.3. Assessment of the plasma exposure–occupancy relationship

The occupancy values obtained from the Lassen plot were entered into an analysis of the curvilinear relationship between drug plasma concentration and pulmonary receptor occupancy. In detail, a weighted non-linear least square curve fit was performed using the following equation:.

(1) $$\mathrm{Occ}=\mathrm{Oc}c_{\max}\times \frac{C_{\;p}}{C_{\;p}+K_{i}}$$

where Occ is occupancy, Occ_max_ is the maximum level of occupancy achievable by tiotropium at the target binding sites detected by [^11^C]VC-002, C_p_ is the average plasma tiotropium concentration during PET and *K*_i_ is the apparent inhibition constant, i.e. the plasma concentration when Occ equals half of Occ_max_.

The model was fitted to the occupancy values estimated obtained from the Lassen plot analysis, yielding estimates of *K*_i_. The maximal occupancy, Occ_max_, was either fixed at 100% or, alternatively, estimated by the fit. The *R*-squared values of the linear fit in the Lassen plot occupancy estimation were used as weighting factors when fitting the occupancy model, i.e., in effect giving more weight to occupancy values that were more certain. The model was fitted in separate analyses to NHP (i.v. administration-based) and human (inhalation based) data, respectively.

### 2.4. Statistics

Processing and computations were performed using the Matlab, version R2014b (www.mathworks.com). The estimation of occupancy using weighted linear least squares provided statistical assessment of the goodness-of-fit, such as the coefficient of determination, *R*^2^, the standard error and a 95% confidence interval for the coefficients. A simple *t*-test was separately performed on the inter-individual mean occupancy values for each group (pretreatment or test-retest) and species (NHP or human). Statistical assessment was performed on the results of the exposure–occupancy model fits: (1) to ascertain the goodness of fit (among others calculating the standard error and 95% confidence interval of fitted parameters), (2) to compare alternative models of the NHP data (by using an *F*-test, Akaike's information criterion values, AIC, and adjusted *R*-squared values), and (3) to compare i.v. administration based (NHP) and inhalation based (human) *K*_i_ estimates (by using two-tailed *t*-tests). In all analyses, the statistical significance (alpha level) was set at *p* < 0.05.

## 3. Results

### 3.1. Study in NHPs

#### 3.1.1. Tiotropium plasma concentration

Three NHPs were included and overall six pretreatment experiments were performed (doses in the range of 0.03 to 1 *µ*g/kg). The measured tiotropium plasma concentrations covered a wide interval, ranging from sub-therapeutic (∼2 pM) to supra-therapeutic levels (∼300 pM). For NHP1, following injection of the low tiotropium dose of 0.03 *µ*g/kg, only the first post-injection sample had a value above the lower limit of quantification (LLOQ). The concentration value used for further analysis was estimated with the help of a reference plasma concentration curve, scaled to the measured peak value (see Supplementary Figure S2).

#### 3.1.2. PET measurements and [^11^C]VC-002 binding in lungs

**Figure 1:**
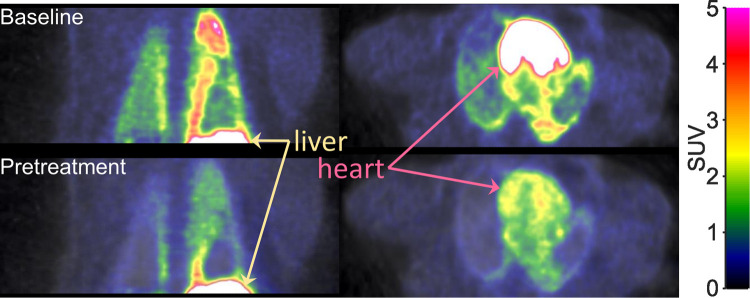
Positron emission tomography (PET) images showing the radioactivity concentration in the upper trunk after i.v. injection of [^11^C]VC-002 in a non-human primate (NHP3). Images show frontal (left column) and horizontal (right column) sections. Uptake at baseline (upper row) and 20 min after a 10-min intravenous infusion (lower row) of 1 *µ*g/kg tiotropium.

Visual inspection of the PET-images obtained after intravenous injection of [^11^C]VC-002 showed that the radioactivity was distributed throughout the lungs (Figure 1). Compared to the radioactivity concentration in lungs, the radioactivity in heart tissue and liver was at a substantially higher level. After administration of tiotropium, there was a conspicuous reduction in radioactivity in lungs and heart, but not liver (Figure 1).

**Figure 2:**
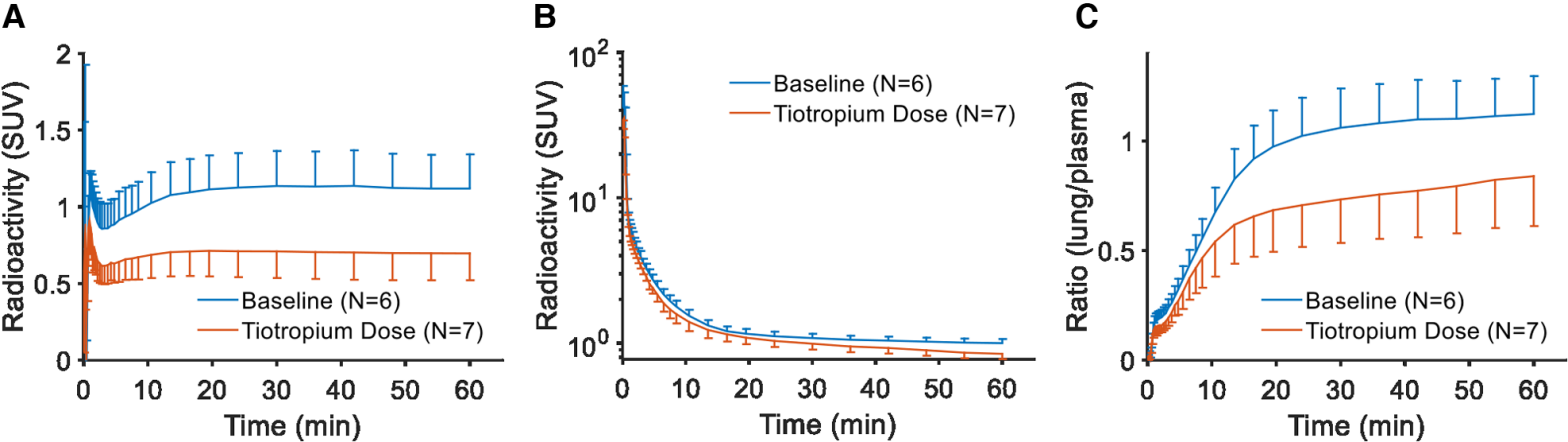
Regional curves for [^11^C]VC-002 in non-human primate. Time-radioactivity curves (TACs) for [^11^C]VC-002 in lung (**A**) and plasma (**B**), as well as the lung-to-plasma ratio (**C**). Curves show inter-individual mean with error bars indicating standard deviation.

The reduction in radioactivity after tiotropium was also evident when inspecting the time curves for the lungs (Figure 2A). For plasma, there was no systematic difference when comparing pretreatment and baseline conditions (Figure 2B). Accordingly, the plots visualizing the ratio of lung tissue to plasma over time showed a marked effect of pretreatment with tiotropium (Figure 2C).

**Figure 3:**
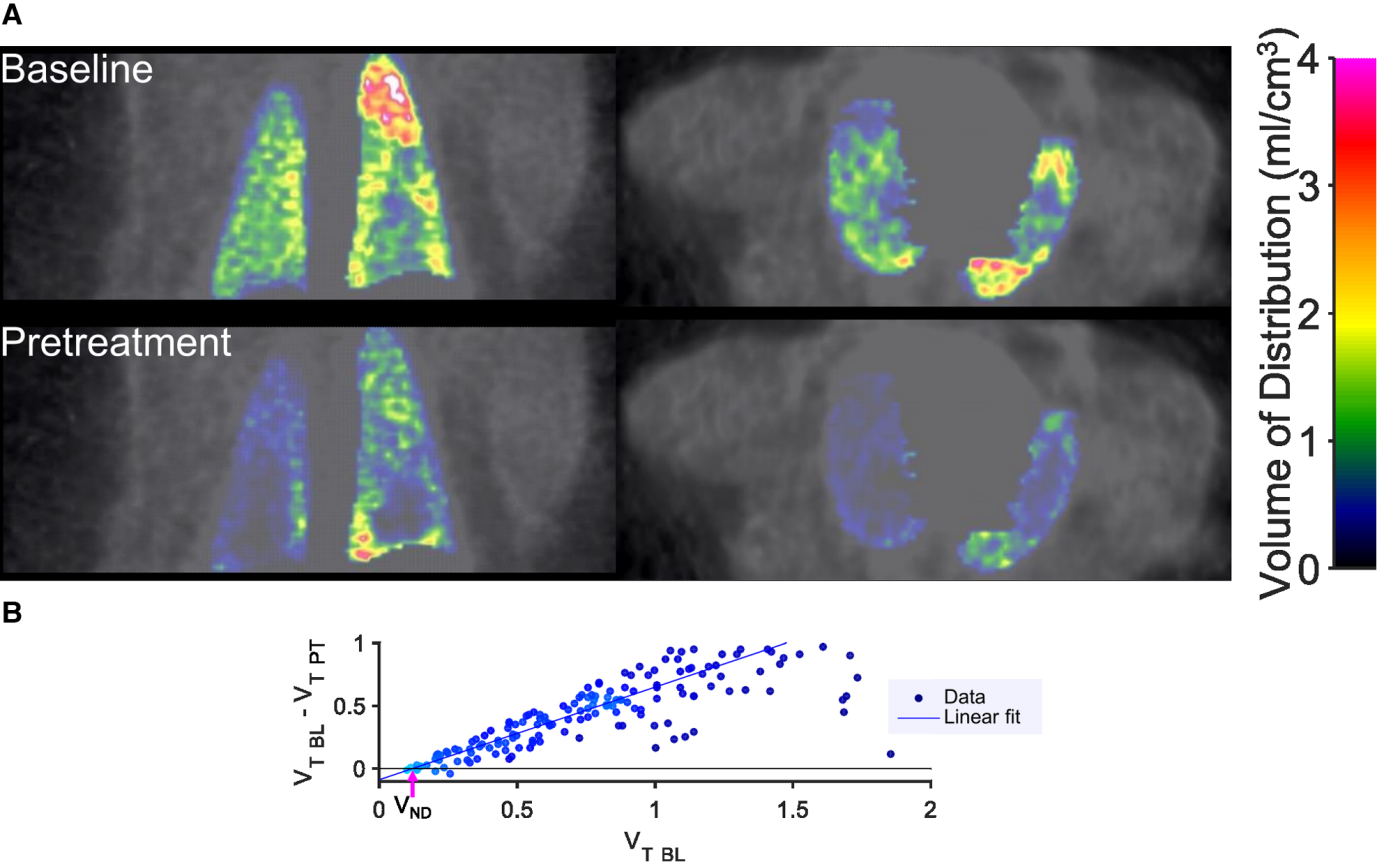
Sample results of quantification of [^11^C]VC-002 binding and occupancy in lung in non-human primate. Parametric images (**A**) of distribution volume V_T_ of [^11^C]VC-002 in lung at baseline (upper row of images) and after tiotropium administration (lower row of images) (individual subject NHP3). Lassen plot data (NHP3) and linear fit used to obtain estimate of muscarinic receptor occupancy in lung (**B**). Straight line indicates linear fit with the x-axis intercept (indicated by the purple arrow) providing the V_ND_ and the slope providing the occupancy estimates, respectively. V_T BL_: V_T_ at baseline, V_T PT_: V_T_ at pretreatment occasion.

Voxel-wise kinetic analysis was performed to estimate the parameter of total radioligand binding (total volume of distribution, V_T_). The resulting parametric images of V_T_ also demonstrated a reduction in total binding in lungs after pretreatment (Figure 3A). The whole lung total binding of [^11^C]VC-002 was reduced in a dose dependent manner up to 63% in NHP following pretreatment but was on average unchanged at test-retest conditions (Table 1).

**Table 1:** [^11^C]VC-002 binding and receptor occupancy estimates.

Species	Experiment	V_T_, baseline Mean ± SD (N)	V_T_, tiotropium or retest Mean ± SD (N)	V_T_ reduction (%) Mean (range)	Occupancy (%) Mean (range)	Occupancy (%) Median	V_ND_ Median
**NHP**	**Tiotropium**	1.6 ± 0.6 (6)	1.1 ± 0.6 (6)	36.4 (−0.4 ‒ 62.7)	48.5 (11.1–77.9)	52.5	0.12
**Human**	**Tiotropium**	1.9 ± 0.5 (6)	1.5 ± 0.4 (10)	17.3 (2.3 ‒ 26.7)	33.3 (6.4–65.0)	33.8	0.41
**NHP**	**Test-retest**	1.8 ± 0.7 (2)	1.7 ± 0.4 (2)	1.0 (−10.8 ‒ 12.8)	7.5 (6.1–8.9)	7.5	N/A
**Human**	**Test-retest**	1.6 ± 0.4 (7)	1.7 ± 0.5 (7)	−5.6 (−28.1 ‒ 3.6)	8.5 (4.0–12.9)	9.7	N/A

Summary statistics of [^11^C]VC-002 binding and estimated receptor occupancy in lungs following tiotropium administration and test-retest experiments in non-human primates and humans. Test-retest data is in 6 subjects from previous publication (16), and subject H4 in this study. Binding was estimated using data-driven estimation of parametric images based on compartmental theory (DEPICT). Lassen plot was used to obtain the estimate of muscarinic receptor occupancy and non-displaceable binding (V_ND_) in lung.

#### 3.1.3. Receptor occupancy in lung tissue

**Figure 4:**
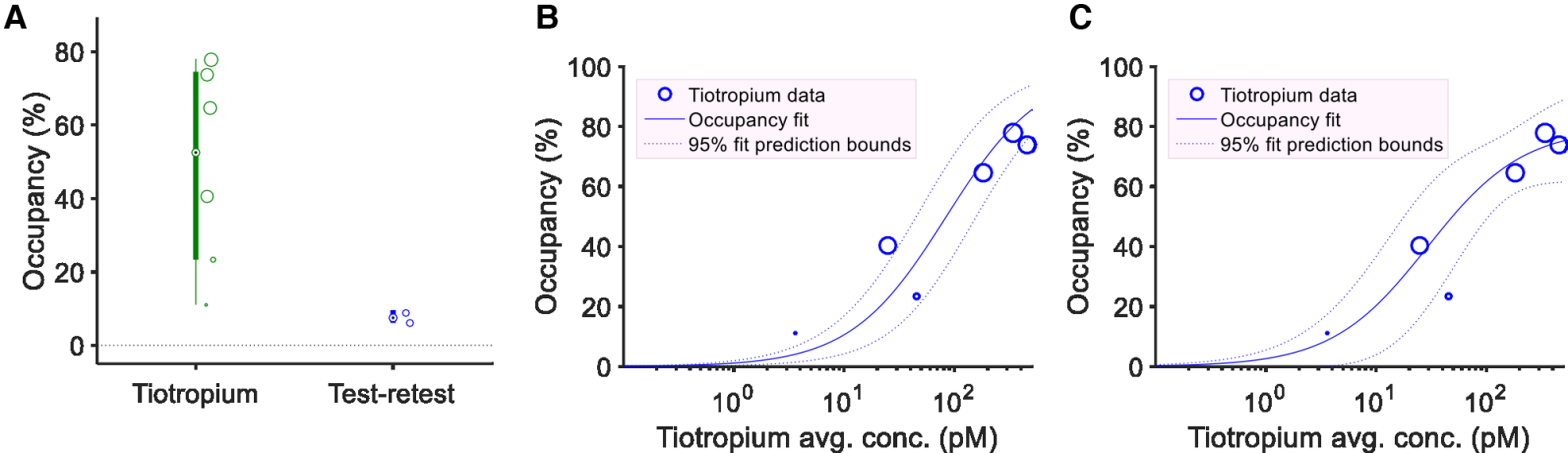
Estimated receptor occupancy and plasma exposure – occupancy relationship in non-human primates. Individual estimates and box plots of estimated receptor occupancy in lungs following six tiotropium administrations or at test-retest conditions (*n* = 2) (**A**). The size of circular markers are proportional to the *R*^2^ value of the linear fit used to obtain the occupancy estimates. Model fit of the plasma exposure – receptor occupancy relationship for tiotropium with either fixed maximum occupancy (Occ_max_) of 100% (**B**) or fitted Occ_max_ (**C**). Circular markers indicate tiotropium scatter point data (average plasma concentration during PET and lung muscarinic occupancy). For the fixed Occ_max_ model (**B**) the estimated *K*_i_ was 84.3 pM (95% CI: 43.2–164.6 pM). For the model with fitted Occ_max_ parameter (**C**) the estimated *K*_i_ was 29.1 pM (95% CI: 11.2–75.5 pM), the estimated Occ_max_ was 79.6% (95% CI: 66.2–93.0%).

The occupancy at the [^11^C]VC-002 binding sites induced by tiotropium was quantified using the Lassen plot (Figure 3B) and varied between 11% and 78%. The values were significantly different from zero (*p* = 0.008, two-tailed *t*-test) (Figure 4A, Table 1). Furthermore, the V_ND_ estimates obtained from the Lassen plots indicated that non-displaceable binding in lung tissue was on average 10% of the baseline total binding (Table 1). The analysis of the two test-retest datasets (NHP1 and NHP2) yielded unbiased Lassen plot estimates of “occupancy” of 8.9% and 6.1%, respectively. These values were used for comparative purposes (Figure 4A, Table 1).

#### 3.1.4. Plasma exposure-receptor occupancy relationship

The occupancy values were plotted vs. the plasma concentration for the six pretreatment measurements in NHP (Figures 4–C). The data could be described by a curvilinear relationship according to Equation 1. *K*_i_, the apparent inhibition constant, which corresponds to the plasma concentration when half of the drug-targeted binding sites are occupied was estimated. Fitting the model with an Occ_max_ parameter fixed at 100% yielded a *K*_i_ estimate of 84.3 pM (95% CI: 43.2–164.6 pM) (Figure 4B). Alternatively, the non-fixed model fit yielded a *K*_i_ estimate of 29.1 pM (95% CI: 11.2–75.5 pM) and an Occ_max_ estimate of 79.6% (95% CI: 66.2–93.0%) (Figure 4C). The non-fixed model fit provided a better description of plasma concentration–occupancy data both visually and according to statistical assessment with the one-tailed *F*-test (*F*-value 9.65, *p*-value: 0.036), or when comparing Akaike's Information Criterion values (AIC: 36.5 for the first model vs. 31.2 for the second model) or the adjusted *R*-squared values of the fitted models (0.59 for the first model vs. 0.85 for the second model).

### 3.2. Study in humans

#### 3.2.1. Subjects and data acquisition

Seven male subjects (H1–H7, mean age 34 years, age range 23–49 years, BMI 19–27 kg/m^2^) were included with a baseline and one or two pretreatment experiments following inhalation of a dose of 18 *µ*g tiotropium (with overall 11 pretreatment measurements). The baseline PET for subject H6 was not possible to evaluate due to severe movement artefacts throughout the experiment. These movement artifacts could not be corrected for by the frame-by-frame correction procedure described in the methods section. Dosing failed for subject H4 in the first pretreatment measurement. The inhaler was by mistake not loaded with tiotropium, thus, effectively resulting in a repeated baseline (retest) measurement. Furthermore, while the inhaler was correctly loaded, yet the plasma analysis was inconclusive, i.e., tiotropium could be detected but the concentration was below LLOQ in subject H5.

#### 3.2.2. Tiotropium plasma concentration

The measured tiotropium concentrations were in the range of 3.6–6.1 pM (4.4 ± 1.0 pM, *N* = 5). When compared to a previously obtained reference curve showing the expected levels for a therapeutic dose (Supplementary Figure S2) the values obtained in the present study were almost 3-fold lower (17).

#### 3.2.3. PET measurements and [^11^C]VC-002 binding in lungs

**Figure 5:**
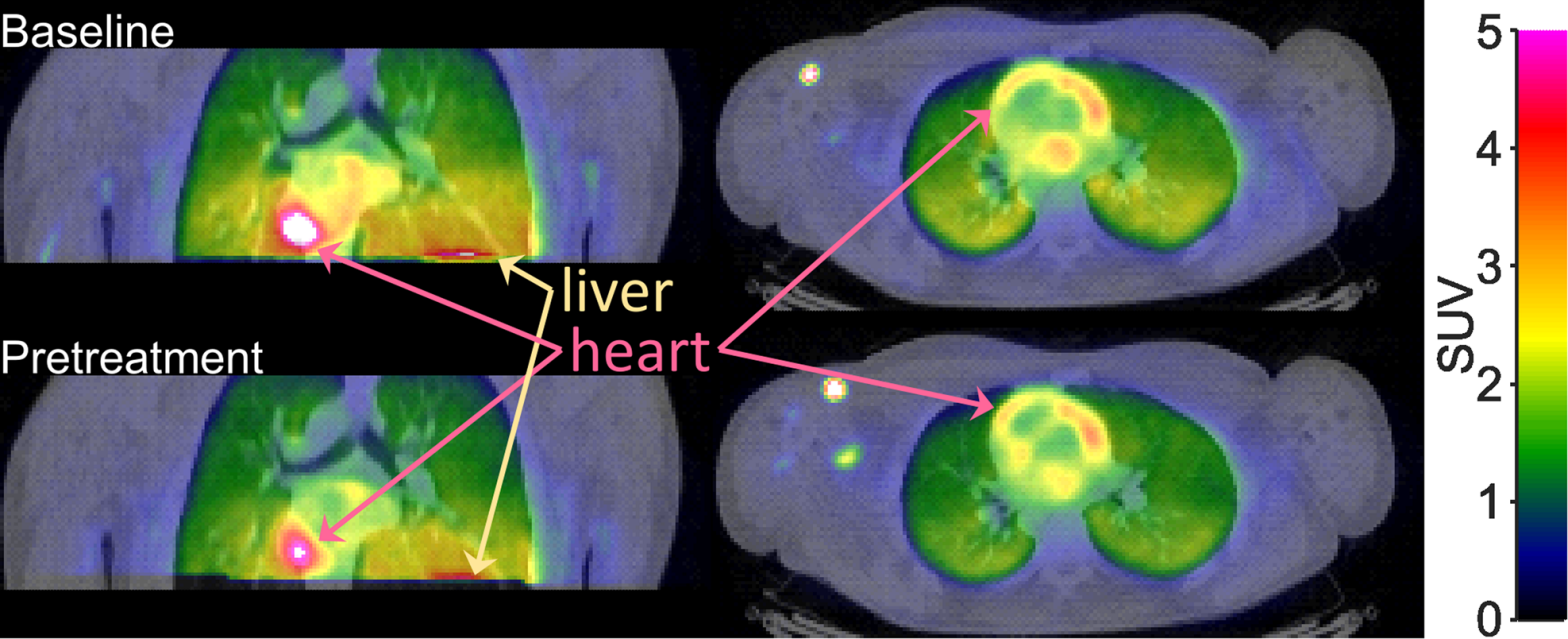
Positron emission tomography (PET) images showing the radioactivity concentration in the upper trunk after i.v. injection of [^11^C]VC-002 in a human subject. Images show frontal (left column) and horizontal (right column) sections through lungs. Data at baseline (upper row) and 2 h after inhalation of 18 *µ*g tiotropium (individual subject H1).

**Figure 6:**
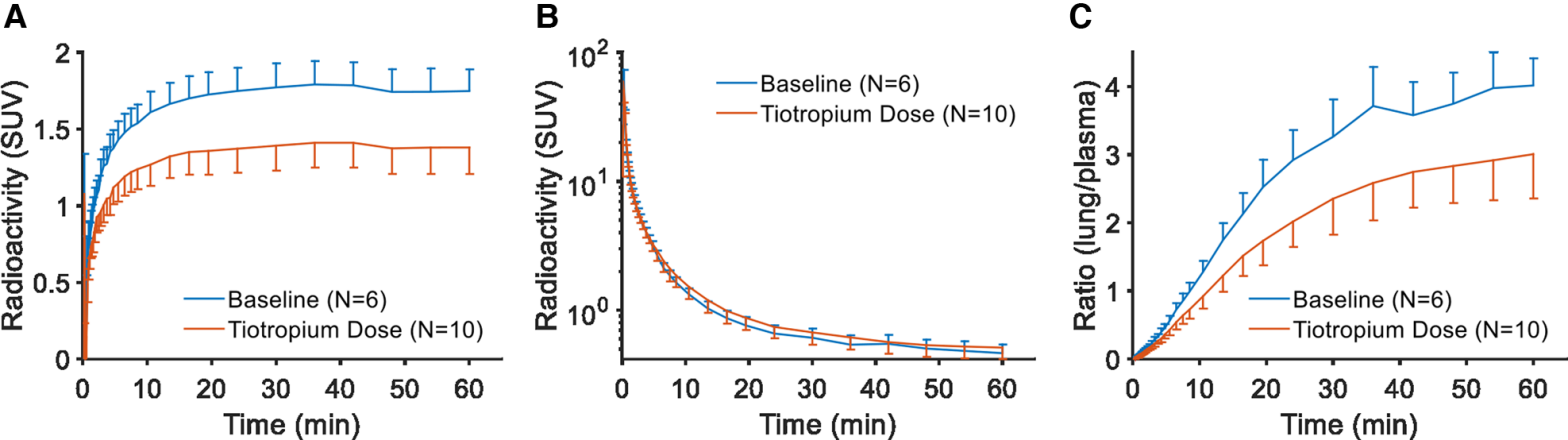
Regional curves for [^11^C]VC-002 in human. Time-radioactivity curves (TACs) for [^11^C]VC-002 in lung (tissue-only) (**A**), plasma (**B**), and lung-to-plasma ratio (**C**). Curves show inter-individual mean with error bars indicating standard deviation.

Following intravenous injection of [^11^C]VC-002, the radioactivity was distributed throughout the lungs (Figure 5) with high levels also in heart and liver. The radioactivity in lungs and heart, but not liver, was reduced following tiotropium inhalation. This reduction could also be observed on the TACs for the lungs (Figure 6A). The plasma TACs did not display an evident difference between baseline and pretreatment measurements (Figure 6B). Accordingly, the plots showing the ratio of radioactivity in lungs to plasma over time indicated a reduction in [^11^C]VC-002 binding after tiotropium inhalation (Figure 6C).

**Figure 7:**
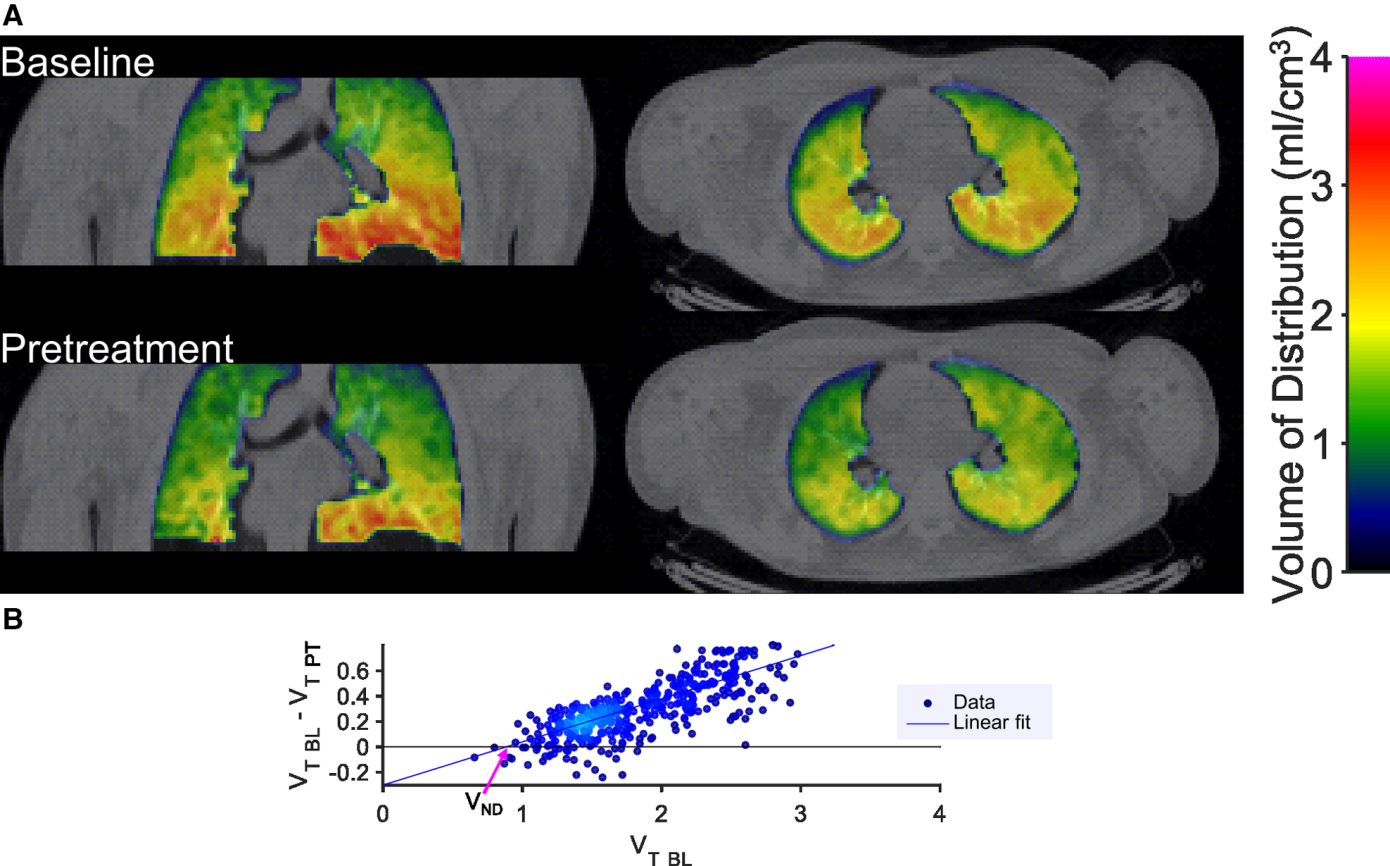
Sample results of quantification of [^11^C]VC-002 binding and occupancy in lung in human. Parametric images (**A**) of distribution volume V_T_ of [^11^C]VC-002 in lung at baseline (upper row of images) and after tiotropium administration (lower row of images) (individual subject H1). Lassen plot data (H1) and linear fit used to obtain estimate of muscarinic receptor occupancy in lung (**B**). Straight line indicates linear fit with the x-axis intercept (indicated by the purple arrow) providing the V_ND_ and the slope providing the occupancy estimates, respectively. V_T BL_: V_T_ at baseline, V_T PT_: V_T_ at pretreatment occasion.

Parametric images of V_T_ also indicated reduced [^11^C]VC-002 binding in lungs after tiotropium inhalation (Figure 7A). The reduction in total binding of [^11^C]VC-002 in lung tissue was 2.3%–26.7% in humans following pretreatment (Table 1). By comparison, on average there was almost no change in whole-lung V_T_ at test-retest when analyzing the 6 subjects from our previous publication and subject H4 in the current dataset (16).

#### 3.2.4. Receptor occupancy in lung tissue

**Figure 8:**
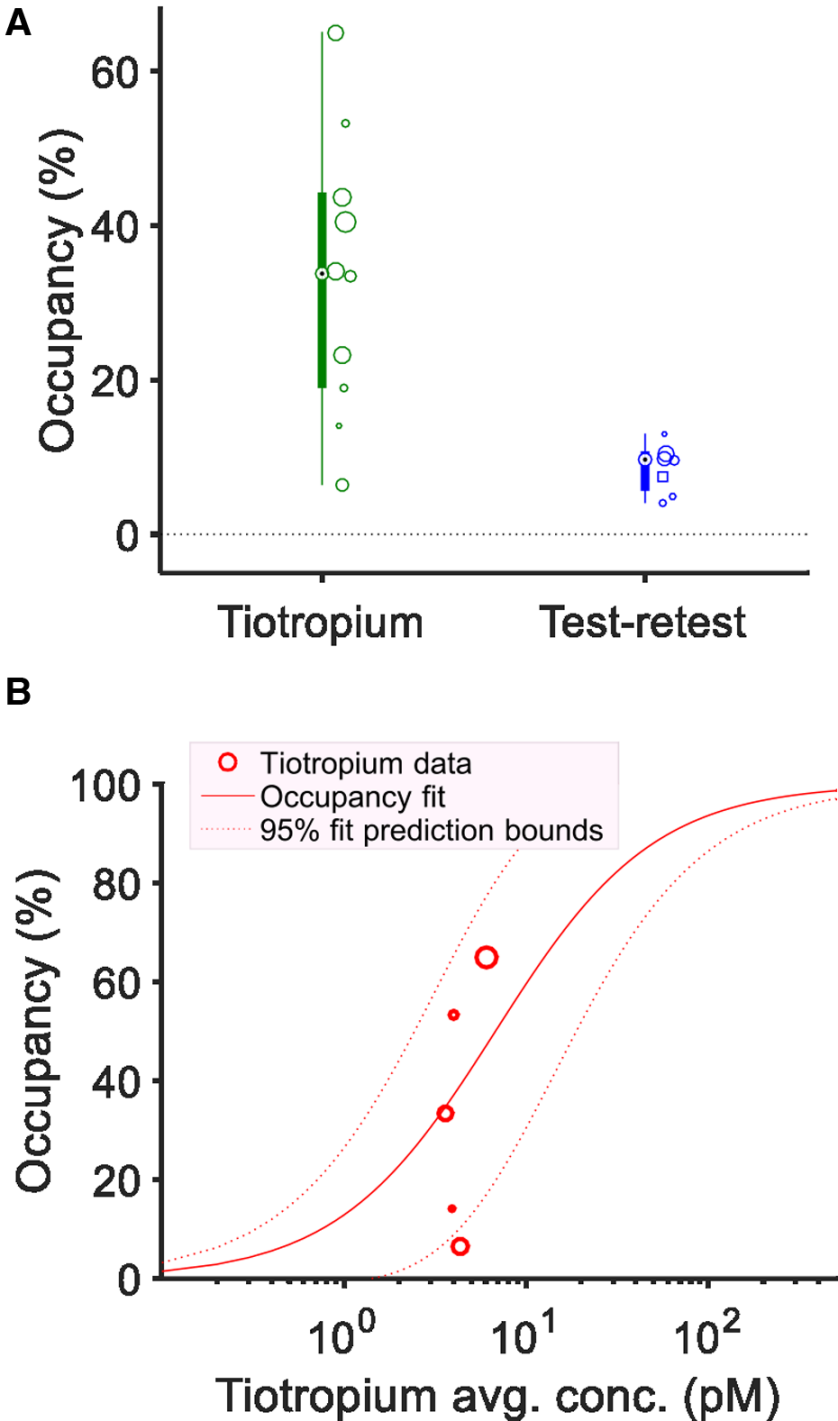
Estimated receptor occupancy and plasma exposure – occupancy relationship in human. Individual estimates and box plots of estimated receptor occupancy in lungs following tiotropium administration or under test-retest conditions (**A**). The size (area) of the individual circular or square markers, showing individual occupancy estimates, are proportional to the *R*^2^ value of the linear fit used to obtain the occupancy, V_ND_ estimates. Source of the test-retest data was either the measurements from the previously published test-retest study (*N* = 6, circular markers) or subject H5 in the current study (square marker). Model fit of the plasma exposure–receptor occupancy relationship for tiotropium providing estimate of apparent inhibition constant (*K*_i_) with fixed maximum occupancy (Occ_max_) of 100% (**B**). Circular markers indicate tiotropium scatter point data (average plasma concentration during PET and lung muscarinic occupancy). The estimated *K*_i_ was 6.8 pM (95% CI: 2.0–22.7 pM).

The Lassen plot was used to estimate mAChR occupancy in lungs as demonstrated for a single subject in Figure 7B. Following tiotropium inhalation, the occupancy estimates in the 10 measurements varied from 6%–65% (Figure 8A, Table 1). They were significantly different from zero (*p* = 0.0003, two-tailed *t*-test). The level of non-displaceable binding (V_ND_) was about 20% of total binding in lung tissue at baseline (Table 1). Lassen-plot based estimates in the test-retest datasets (i.e., six subjects from the previous publication and H5 from the current dataset) were on average 8.5%, and also significantly different from zero (*p* = 0.0004, two-tailed *t*-test (Table 1) (16). Furthermore, the occupancy estimates in the pretreatment data were significantly different from those in the test-retest dataset (*p* = 0.003, two-tailed *t*-test, 95% CI of the difference in occupancy: was 9.9%–39.7%).

#### 3.2.5. Plasma exposure–occupancy relationship

The tiotropium plasma concentration–occupancy data in human fell in a relatively narrow plasma exposure range with a large variation in occupancy values (Figure 8B). As such, only the exposure–occupancy model with a fixed Occ_max_ of 100% could be readily applied, which yielded an affinity (*K*_i_) estimate of 6.8 pM (95% CI: 2.0–22.7 pM) (Figure 8B). Two-tailed *t*-tests comparing the human (inhalation-based) *K*_i_ estimate to those obtained in NHP (following i.v. administration, Figure 4B,C) indicated a significant difference between the two administration routes and species (*p*-values below 0.0003).

## 4. Discussion

In a translational setting we demonstrated an evident effect of the long-acting muscarinic antagonist tiotropium on [^11^C]VC-002 binding to muscarinic receptors in lungs in both NHPs and humans. The study shows that it is possible to detect and quantify receptor occupancy in lungs following drug administration. The methodology has potential not only for dose finding and comparison of drug formulations in future applied studies, but also for evaluating changes in lung receptor distribution during disease or in response to therapy.

A wide dose range of tiotropium was examined in NHPs. The binding was reduced in a saturable dose-dependent fashion and at the largest dose the total binding (V_T_) in the whole lung was reduced by about two thirds (Table 1). This result suggests that a major proportion of the total [^11^C]VC-002 binding in lung represents specific binding to mAchR, whereas a minor proportion represents a background of free and non-specific [^11^C]VC-002 binding. Furthermore, an occupancy effect was detected also in heart but not in liver. This finding agrees with the known distribution of muscarinic receptors. As such, the strong, non-competitive signal from the liver likely represents hepatobiliary elimination.

The Lassen plot analysis of occupancy provided direct estimates of the reduction of specific binding, i.e., the mAchR occupancy. The occupancy reached 80% for the highest tiotropium dose (1.0 *µ*g/kg). Interestingly, the saturation curve analysis of the exposure – occupancy data in NHPs indicated that the projected maximum occupancy possibly achievable by tiotropium at the binding sites recognized by [^11^C]VC-002 was less than 100% (Occ_max _= 80%). The composition of the remaining, non-displaceable portion is however not clear. Besides representing free and non-specific binding it has to be considered that both [^11^C]VC-002 and tiotropium are non-selective compounds with high affinity for M1-M3 muscarinic subtypes (13, 17). However, both compounds have different affinity profiles across the M1-M3 receptors, thus, tiotropium may not fully block all [^11^C]VC-002 binding and by that contribute to the estimated non-specific compartment.

The relationship between lung occupancy and tiotropium plasma concentration was evaluated according to the classical occupancy model. The blood (plasma) compartment is a true input to the lung tissue only in case of intravenous administration and is instead an “output”, in case of drug inhalation. It has previously been demonstrated that there is a rigorous relationship between inhalation dosing and the observed time profile of drug concentration in plasma, with a possible steady-state between lung parenchyma and plasma from 30 to 60 min post inhalation, thus supporting the use of the classical occupancy model (17).

In humans, high doses of tiotropium could not be examined for safety reasons. In the present study, only a single, therapeutic dose of 18 *µ*g was evaluated and the reduction of total [^11^C]VC-002 binding was small when compared to the effect of high dose i.v. administration of tiotropium in NHP. Only moderate lung occupancy was detected with estimated values mostly below 50% (range 6%–65%)., a potential limitation is that the study was performed in a group of healthy subjects, who had no previous experience with inhalation apart from a short training session. As such, inhalation of tiotropium may have been suboptimal, which could explain the approximately 3-fold lower plasma exposure than expected as defined for a therapeutic dose (see Supplementary Figure S2). Thus, the moderate mAChR occupancy may have been due to tiotropium underexposure.

Importantly, the present application of the occupancy model to the human data is only tentative due to the limited spread of plasma tiotropium concentrations and the large variation of estimated occupancy values. In the future, the relationship between drug concentration and occupancy has to be examined over a larger dose range in experienced inhalation users (patients). In contrast, occupancy values appeared to be less variable in NHP when accounting for the occupancy – exposure relationship (see the closeness of most individual data points to the model curve in Figures 4B,C). Worth noting is that there was a significant difference between the estimated *in vivo* affinity in the two species with roughly one order of magnitude larger NHP *K*_i_ values (29.1 or 84.3 pM depending on model) than the human *K*_i_ value (6.8 pM). Species differences may play a role here, though the muscarinic receptor protein sequences are highly homogeneous between NHPs and humans. In detail, there are only 3 (out of 466) amino-acid differences for the M2 receptor and 9 (out of 590) differences for the M3 receptor, all only in non-ligand-binding regions of the proteins (22–25). A more likely explanation is thus provided by the differences in the route of administration, i.e., intravenous infusion in NHP vs. inhalation in human. Importantly, prior work, executed in NHP using ipratropium has demonstrated a similar difference in apparent potency between the two administration routes only in lung but not in other mAchR-rich organs (15). This difference demonstrates the targeting effect of inhalation, which translates to the clinical benefit of achieving therapeutic drug effect in lungs while minimizing side effects due to muscarinic blockade in other organs such as the heart. Furthermore, our findings of sub-nanomolar *K*_i_ values for tiotropium both in NHPs and in humans are in line with *in vitro K*_d_, *K*_i_ values for binding to human muscarinic receptor preparations (*K*_d_: 120-270 pM), isolated and homogenized rat lung tissue (*K*_i_: 130 pM) and human airway smooth muscle (*K*_d_: 38.6 pM) (26–28).

A further aspect of characterizing drug target engagement is the time course of receptor occupancy following inhalation. Detailed evaluation of the time course was beyond the scope of the present work. Anyhow, in a preliminary attempt we examined the level of occupancy in a one-hour-long acquisition window starting at 30 min and 2 h post inhalation, respectively. The results indicated detectable lung occupancy of similar magnitude at both time intervals, which is in line with the known long half-life of dissociation of tiotropium from binding sites (26). When applying the classical occupancy model in future studies to evaluate multiple doses, care should be taken to account for the timing between inhalation and measurement of occupancy. Ideally, PET experiments with a displacement paradigm, i.e., drug inhalation during an ongoing [^11^C]VC-002 PET acquisition, could be used to assess the early and detailed time course of lung occupancy. If the off-rate of specific binding of [^11^C]VC-002 is indeed 0.05–0.1 1/min, as previous data hints at, then a substantial fraction of specific binding may be displaced in such experiments within the typical 1–2 h time-frame of a PET measurement (16).

Subject movement during PET acquisition poses a challenge to evaluating radioligand binding and thus drug effect in lungs. In the present work, a previously described frame-by-frame correction approach was used to address this issue (16). While yielding acceptable results in most cases, the method has a limitation when subjects have substantial intra-frame movement which cannot be corrected using this scheme, such as it happened in one case in the current study (baseline PET for subject H6).

Another limitation was that fractional air content inhomogeneities were not corrected for in the results, chiefly because NHP had no CT images necessary for the correction. Theoretically, air-content related inhomogeneities may lead to violation of the underlying assumptions of the Lassen plot, such as an even level of non-displaceable binding across the organ. However, in the present study the assumption appeared to be supported by the predominantly linear distribution of the Lassen plot across scatter points. Nonetheless, incorporating air-content correction could prove to be beneficial in occupancy studies in patients who have more extreme lung compositional inhomogeneities such as fibrosis and emphysema.

New targets for respiratory drug development have been identified in current research on the pathophysiology and diagnostics of chronic pulmonary disorders. The list of emerging targets include integrin, lysophosphatidic acid receptor, FAPI, adrenomedullin receptors and, CCR2, among others (29–36). Such drug discovery and development projects might likely benefit from the novel methodology presented here.

In conclusion, the present study demonstrated that [^11^C]VC-002 binds specifically to mAChRs in the lungs such that it allows for the detection and quantification of receptor occupancy following administration of intravenously injected as well as inhaled muscarinic antagonist drugs. The methodology has potential for dose finding and comparison of drug formulations in future applied studies.

## Data Availability

The original contributions presented in the study are included in the article/**Supplementary Materials**, further inquiries can be directed to the corresponding author/s.

## References

[1] Matera MG Page CP Calzetta L Rogliani P Cazzola M. Pharmacology and therapeutics of bronchodilators revisited. Pharmacol Rev. (2020) 72(1):218–52. 10.1124/pr.119.018150 31848208

[2] Maia IS Pincelli MP Leite VF Amadera J Buehler AM. Long-acting muscarinic antagonists vs. Long-acting *β* 2 agonists in COPD exacerbations: a systematic review and meta-analysis. J Bras Pneumol. (2017) 43(4):302–12. 10.1590/s1806-37562016000000287 28767773 PMC5687968

[3] Melani AS. Long-acting muscarinic antagonists. Expert Rev Clin Pharmacol. (2015) 8(4):479–501. 10.1586/17512433.2015.1058154 26109098

[4] Lal C Khan A. Emerging treatments for COPD: evidence to date on revefenacin. COPD: J Chronic Obstruct Pulm Dis. (2020) 17(1):112–9. 10.1080/15412555.2019.1702010 PMC9676114 31833419

[5] Bäckman P Adelmann H Petersson G Jones CB. Advances in inhaled technologies: understanding the therapeutic challenge, predicting clinical performance, and designing the optimal inhaled product. Clin Pharmacol Ther. (2014) 95(5):509–20. 10.1038/clpt.2014.27 24503626

[6] Farde L Wiesel FA Halldin C Sedvall G. Central D2-dopamine receptor occupancy in schizophrenic patients treated with antipsychotic drugs. Arch Gen Psychiatry. (1988 1) 45(1):71–6. 10.1001/archpsyc.1988.01800250087012 2892477

[7] Waarde A van. Measuring receptor occupancy with PET. Curr Pharm Des. (2000) 6(16):1593–610. 10.2174/1381612003398951 10974155

[8] Lee CM Farde L. Using positron emission tomography to facilitate CNS drug development. Trends Pharmacol Sci. (2006) 27(6):310–6. 10.1016/j.tips.2006.04.004 16678917

[9] Varnäs K Nyberg S Karlsson P Pierson ME Kågedal M Cselényi Z Dose-dependent binding of AZD3783 to brain 5-HT1B receptors in non-human primates and human subjects: a positron emission tomography study with [11C]AZ10419369. Psychopharmacology. (2011) 213(2–3):533–45. 10.1007/s00213-011-2165-z 21234549

[10] Nord M Nyberg S Brogren J Jucaite A Halldin C Farde L. Comparison of D2 dopamine receptor occupancy after oral administration of quetiapine fumarate immediate-release and extended-release formulations in healthy subjects. Int J Neuropsychopharmacol. (2011) 14(10):1357–66. 10.1017/S1461145711000514 21477416 PMC3198174

[11] Jucaite A Takano A Boström E Jostell KG Stenkrona P Halldin C AZD5213: a novel histamine H3 receptor antagonist permitting high daytime and low nocturnal H3 receptor occupancy, a PET study in human subjects. Int J Neuropsychopharmacol. (2013) 16(6):1231–9. 10.1017/S1461145712001411 23217964

[12] Jucaite A Cselényi Z Lappalainen J McCarthy DJ Lee CM Nyberg S GABAA Receptor occupancy by subtype selective GABAA*α*2,3 modulators: pET studies in humans. Psychopharmacology. (2017) 234(4):707–16. 10.1007/s00213-016-4506-4 28013354 PMC5263201

[13] Visser TJ van Waarde A Jansen TJ Visser GM van der Mark TW Kraan J Stereoselective synthesis and biodistribution of potent [11C]-labeled antagonists for positron emission tomography imaging of muscarinic receptors in the airways. J Med Chem. (1997) 40(1):117–24. 10.1021/jm960374w 9016336

[14] Visser TJ van Waarde A van der Mark TW Kraan J Ensing K Willemsen AT Detection of muscarinic receptors in the human lung using PET. J Nucl Med. (1999) 40(8):1270–6. 10450677

[15] Schou M Ewing P Cselenyi Z Fridén M Takano A Halldin C Pulmonary PET imaging confirms preferential lung target occupancy of an inhaled bronchodilator. EJNMMI Res. (2019) 9(1):9. 10.1186/s13550-019-0479-8 30694407 PMC6890867

[16] Cselényi Z Jucaite A Kristensson C Stenkrona P Ewing P Varrone A Quantification and reliability of [11C]VC - 002 binding to muscarinic acetylcholine receptors in the human lung — a test-retest PET study in control subjects. EJNMMI Res. (2020) 10(1):59. 10.1186/s13550-020-00634-0 32495011 PMC7270393

[17] Hohlfeld JM Sharma A van Noord JA Cornelissen PJG Derom E Towse L Pharmacokinetics and pharmacodynamics of tiotropium solution and tiotropium powder in chronic obstructive pulmonary disease. J Clin Pharmacol. (2014) 54(4):405–14. 10.1002/jcph.215 24165906 PMC4263162

[18] Gunn RN Gunn SR Turkheimer FE Aston JAD Cunningham VJ. Positron emission tomography compartmental models: a basis pursuit strategy for kinetic modeling. J Cereb Blood Flow Metab. (2002) 22(12):1425–39. 10.1097/01.wcb.0000045042.03034.42 12468888

[19] Lassen NA Bartenstein PA Lammertsma AA Prevett MC Turton DR Luthra SK Benzodiazepine receptor quantification in vivo in humans using [11C]flumazenil and PET: application of the steady-state principle. J Cereb Blood Flow Metab. (1995) 15(1):152–65. 10.1038/jcbfm.1995.17 7798333

[20] Cunningham VJ Rabiner EA Slifstein M Laruelle M Gunn RN. Measuring drug occupancy in the absence of a reference region: the lassen plot re-visited. J Cereb Blood Flow Metab. (2010) 30(1):46–50. 10.1038/jcbfm.2009.190 19738632 PMC2949110

[21] Ichise M Toyama H Innis RB Carson RE. Strategies to improve neuroreceptor parameter estimation by linear regression analysis. J Cereb Blood Flow Metab. (2002) 22(10):1271–81. 10.1097/01.WCB.0000038000.34930.4E 12368666

[22] https://www.ncbi.nlm.nih.gov/protein/NP_001365901.1.

[23] https://www.ncbi.nlm.nih.gov/protein/XP_014990471.1.

[24] https://www.ncbi.nlm.nih.gov/protein/NP_001362913.1.

[25] https://www.ncbi.nlm.nih.gov/protein/NP_001244669.1.

[26] Disse B Reichl R Speck G Traunecker W Rominger KL Hammer R. Ba 679 BR, A novel long-acting anticholinergic bronchodilator. Life Sci. (1993) 52(5):537–44. 10.1016/0024-3205(93)90312-Q 8441333

[27] Ogoda M Niiya R Koshika T Yamada S. Comparative characterization of lung muscarinic receptor binding after intratracheal administration of tiotropium, ipratropium, and glycopyrrolate. J Pharmacol Sci. (2011) 115(3):374–82. 10.1254/jphs.10311FP 21358117

[28] Haddad EB Mak JC Barnes PJ. Characterization of [3H]ba 679 BR, a slowly dissociating muscarinic antagonist, in human lung: radioligand binding and autoradiographic mapping. Mol Pharmacol. (1994) 45(5):899–907. 8190106

[29] Martinez LMA Harel F Létourneau M Finnerty V Fournier A Dupuis J SPECT And PET imaging of adrenomedullin receptors: a promising strategy for studying pulmonary vascular diseases. Am J Nucl Med Mol Imaging. (2019) 9(5):203–15. 31772819 PMC6872478

[30] Niemeijer AN Leung D Huisman MC Bahce I Hoekstra OS van Dongen GAM Whole body PD-1 and PD-L1 positron emission tomography in patients with non-small-cell lung cancer. Nat Commun. (2018) 9(1):4664. 10.1038/s41467-018-07131-y 30405135 PMC6220188

[31] Maher TM Simpson JK Porter JC Wilson FJ Chan R Eames R A positron emission tomography imaging study to confirm target engagement in the lungs of patients with idiopathic pulmonary fibrosis following a single dose of a novel inhaled *α*v*β*6 integrin inhibitor. Respir Res. (2020) 21(1):75. 10.1186/s12931-020-01339-7 32216814 PMC7099768

[32] Bergmann C Distler JHW Treutlein C Tascilar K Müller AT Atzinger A 68Ga-FAPI-04 PET-CT for molecular assessment of fibroblast activation and risk evaluation in systemic sclerosis-associated interstitial lung disease: a single-centre, pilot study. Lancet Rheumatol. (2021) 3(3):e185–94. 10.1016/S2665-9913(20)30421-5 38279381

[33] Brody SL Gunsten SP Luehmann HP Sultan DH Hoelscher M Heo GS Chemokine receptor 2–targeted molecular imaging in pulmonary fibrosis. A clinical trial. Am J Respir Crit Care Med. (2021) 203(1):78–89. 10.1164/rccm.202004-1132OC 32673071 PMC7781144

[34] Gallezot JD Nabulsi NB Holden D Lin SF Labaree D Ropchan J Evaluation of the lysophosphatidic acid receptor type 1 radioligand 11C-BMT-136088 for lung imaging in rhesus monkeys. J Nucl Med. (2018) 59(2):327–33. 10.2967/jnumed.117.195073 28864634

[35] Harris RS Schuster DP. Visualizing lung function with positron emission tomography. J Appl Physiol. (2007) 102(1):448–58. 10.1152/japplphysiol.00763.2006 17038490

[36] Lukey PT Coello C Gunn R Parker C Wilson FJ Saleem A Clinical quantification of the integrin *α*v*β*6 by [18F]FB-A20FMDV2 positron emission tomography in healthy and fibrotic human lung (PETAL study). Eur J Nucl Med Mol Imaging. (2020) 47(4):967–79. 10.1007/s00259-019-04586-z 31814068 PMC7075837

