# Supplementary material for: Proof of lung muscarinic receptor occupancy by tiotropium: Translational Positron Emission Tomography studies in non-human primates and humans
Source: Front Nucl Med. 2023 Jan 18;2:1080005. doi: 10.3389/fnume.2022.1080005 (PMC11440881; doi:10.3389/fnume.2022.1080005)
Supplement: Supplementary file 1 [file Datasheet1.pdf]

Supplementary Material

1 Supplementary Figures

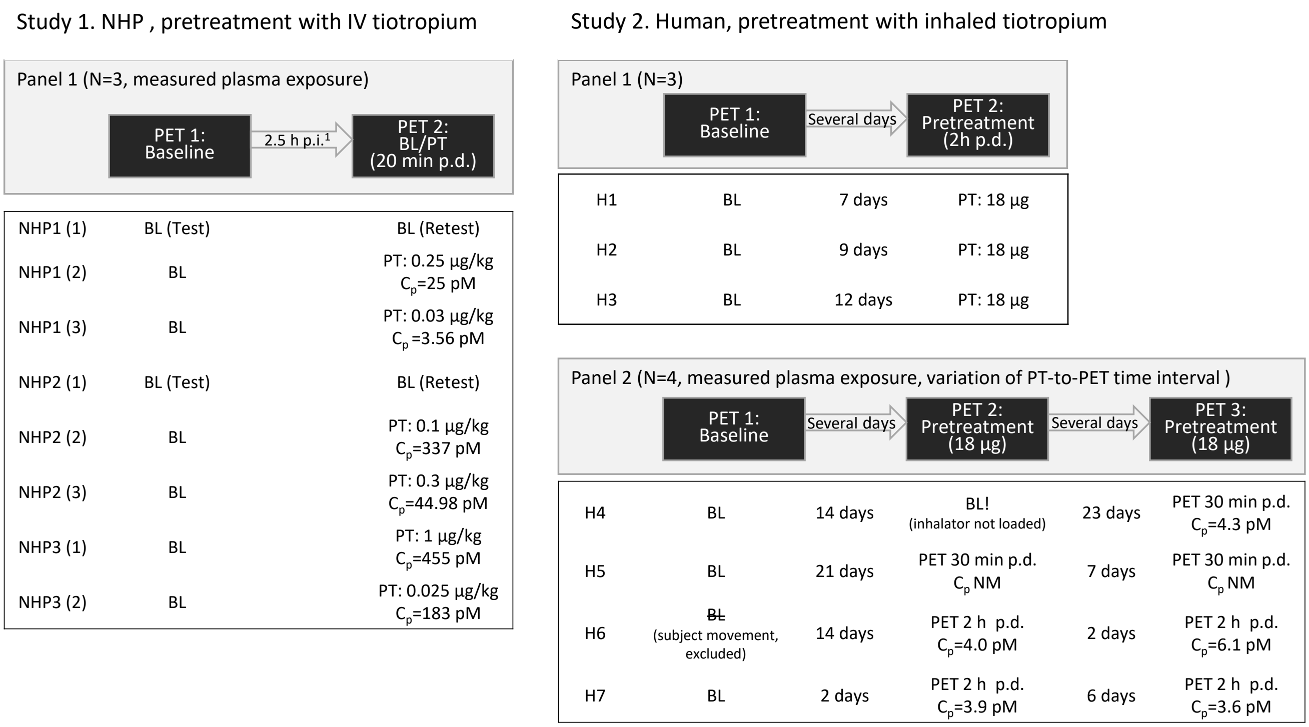

Supplementary Figure 1. Study design and actual measurements. Abbreviations: p.i., post injection of radioligand for PET 1; BL, baseline measurement; PT, pretreatment measurement; p.d., post (tiotropium) dose; C_p, tiotropium plasma concentration (exposure); NM, not measurable (sample below lower limit of quantification).

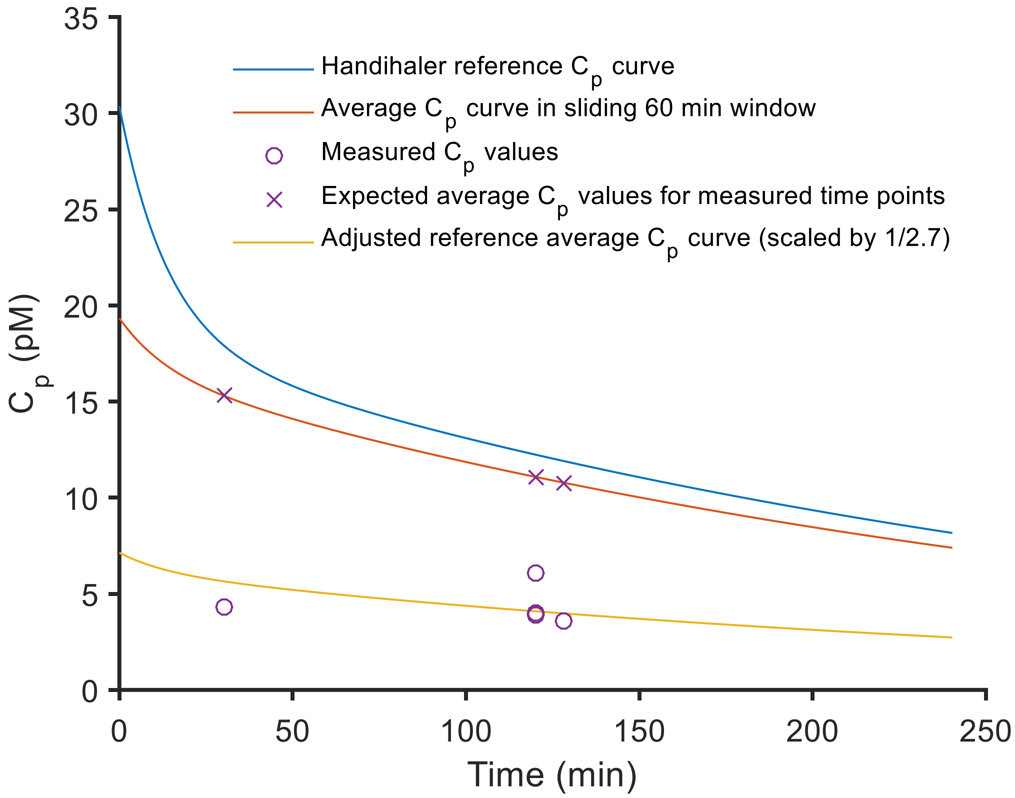

Supplementary Figure 2. Comparison of measured tiotropium plasma concentration (Cp) data to expected levels. The tiotropium HandiHaler® reference curve (for 18 µm dose, (*17*)) were used to determine the expected average plasma concentration during the pretreatment PET experiments for the purpose of comparing them to measured data. In detail, the following steps were taken to determine expected plasma concentration values and visualize the observed trend. 1. The HandiHaler® reference curve provides information on instantaneous concentration at each timepoint (blue curve). Accordingly, the average plasma concentration (C_p) in a moving 60-minute time window was calculated for each timepoint (orange curve), i.e. the area under the curve was obtained for the 60-minute time window starting at each timepoint and was divided by 60. 2. The expected average C_p was obtained for each PET experiment with a measured average plasma concentration by reading off the Y-axis value of the average C_p curve for the timepoint when the PET experiment started in minutes following inhalation (purple x markers). 3. The reference average C_p curve was adjusted (scaled) using the ratio of the mean of the measured average C_p values (purple circle markers) and the mean of the corresponding reference average C_p values. The scaling factor was 1/2.7, i.e. the *de facto* achieved plasma concentrations were almost 3-fold below the expected therapeutic levels.
